# Selected serum microRNA, abdominal aortic calcification and risk of osteoporotic fracture

**DOI:** 10.1371/journal.pone.0216947

**Published:** 2019-05-14

**Authors:** Marie-Eva Pickering, Marjorie Millet, Jean-Charles Rousseau, Martine Croset, Pawel Szulc, Olivier Borel, Elisabeth Sornay Rendu, Roland Chapurlat

**Affiliations:** 1 Service de Rhumatologie et Pathologie Osseuse, Hôpital E Herriot, HCL, Lyon, France; 2 Inserm UMR 1033, Lyon, France; Universite de Nantes, FRANCE

## Abstract

**Context:**

MicroRNA (miRNA) regulate post-transcriptionally the expression of osteogenesis and angiogenesis associated genes and emerge as potential non-invasive biomarkers in vascular and bone diseases. Severe abdominal aortic calcification (AAC) is associated with higher risk of cardiovascular event and of fragility fracture.

**Objective:**

To identify miRNA linked to the aggravation of AAC and to incident osteoporotic fracture.

**Design:**

Postmenopausal women (>50 years) with available serum at inclusion and data for each outcome (Kauppila score and incident fracture) were selected from the OFELY prospective cohort. We conducted a case-control study in 434 age-matched women, 50% with incident osteoporotic fracture over 20 years of follow-up and a second study in 183 women to explore AAC over 17 years.

**Methods:**

Serum expression of three miRNA involved in vascular calcification and bone turnover regulation (miRs-26a-5p,-34a-5p, and -223-5p) was quantified at baseline by TaqMan Advanced miRNA technology and expressed by relative quantification. Outcomes were the association of miRNA levels with (1) incident osteoporotic fractures during 20 years, (2) AAC aggravation during 17 years.

**Results:**

MiRNA level was not associated with incident fractures (miR-26a-5p: 1.06 vs 0.99, p = 0.07; miR-34a-5p: 1.15 vs 1.26, p = 0.35; miR-223a-5p: 1.01 vs 1.05, p = 0.32). 93 women had an increase in Kauppila score over 17 years while 90 did not. None of the miRNAs was associated with an aggravation in AAC (miR-26a-5p: 1.09 vs 1.10, p = 0.95; miR-34a-5p: 0.78 vs 0.73, p = 0.90; miR-223-5p: 0.97 vs 0.78, p = 0.11).

**Conclusions:**

Circulating miR-26a-5p, -34a-5p and -223-5p are not significantly associated with incident fracture and AAC aggravation.

## Introduction

Although osteoporosis and cardiovascular disease are traditionally viewed as separate disease entities, increased cardiovascular risk is significantly associated with the risk of fragility fracture in hips and vertebrae [1–3]. Both conditions share an increase in prevalence with aging and other risk factors such as menopause, smoking, alcohol consumption and low physical activity [4,5].

Abdominal aortic calcification (AAC) is assessed by semi-quantitative score on spine radiographs and spine scans obtained by Dual-Energy X-ray Absorptiometry (DXA). Severe AAC is associated with a higher risk of major cardiovascular event [5–9]. The fine regulation of arterial vessel calcification involves hormones, cytokines, calcium deposal, other bone remodeling factors, and the differentiation of vascular smooth muscle cells to osteoblast-like cells [6–10]. Severe AAC reflecting poor cardiovascular health status and disturbed blood flow in the vascular system is also associated with lower bone mineral density (BMD), faster bone loss and a higher risk of major fragility fracture [11–14]. This fracture risk remains increased after adjustment for BMD and other potential risk factors. Severe AAC is also related to increase in vertebral fracture in older men [15].

Biological factors such as bone morphogenetic proteins, osteoprotegerin, receptor activator of nuclear factor κB ligand, parathyroid hormone, phosphate, oxidized lipids and vitamins D and K are altered in both diseases [16]. A better knowledge of the mechanisms underlying the association between AAC and fracture risk would lead to the identification of biological markers in individuals who are both at higher risk of cardiovascular event and osteoporotic fracture.

MicroRNAs (miRNAs) are small endogenous regulatory RNAs that influence many physiological and pathophysiological processes by acting as epigenetic key actors in the regulation of gene expression [17]. Numerous studies have reported the miRNA-mediated regulation of bone development and homeostasis through their activity in osteoblastogenesis and osteoclastogenesis [18,19]. Some specific miRNAs that are actors in bone micro-architecture and fragility are also involved in the pathogenesis of cardiovascular diseases, including angiogenesis and the vascular calcification process [20–23]. Thus, miRNAs may regulate bone disease (osteoporosis) and vascular calcification, two processes that share some pathogenetic mechanisms. Furthermore, the level of circulating miRNAs in biofluids would reflect the alteration in the patient tissues. In osteoporotic patients, miRNA level in serum has been associated to alterations of bone metabolism, decreased bone mass and risk of fractures [24,25]. Circulating miRNAs appear as promising non-invasive biomarkers [26]. In regard to their short length and association with proteins and exosomes, miRNAs resist to RNAse digestion and to multiple freeze-thaw cycles and present elevated stability in frozen samples across the years [27].

We conducted the study to clarify the link between cardiovascular risk and osteoporosis in a well-characterized cohort of women and to find a common determinant to these two pathologies. Cardiovascular risk and osteoporosis are assessed by factors easy to use in clinical practice. The purpose of this study was to find out whether the levels of specific circulating miRNAs might provide information on the cardiovascular risk, evaluated with CAA, and the incidence of osteoporotic fracture in postmenopausal women over 20 years of follow-up. Three miRNAs (miRs-26a-5p, 34a-5p, and 223-5p) have been selected for their previously reported activity on both the cardiovascular and bone systems. Their potential association with the progression of AAC and the risk of fractures has been investigated by analyzing miRNA concentrations in the serum of patients from a nested case-control analysis of the prospective OFELY cohort.

## Material and methods

### Study design and subjects

**Fig 1:**
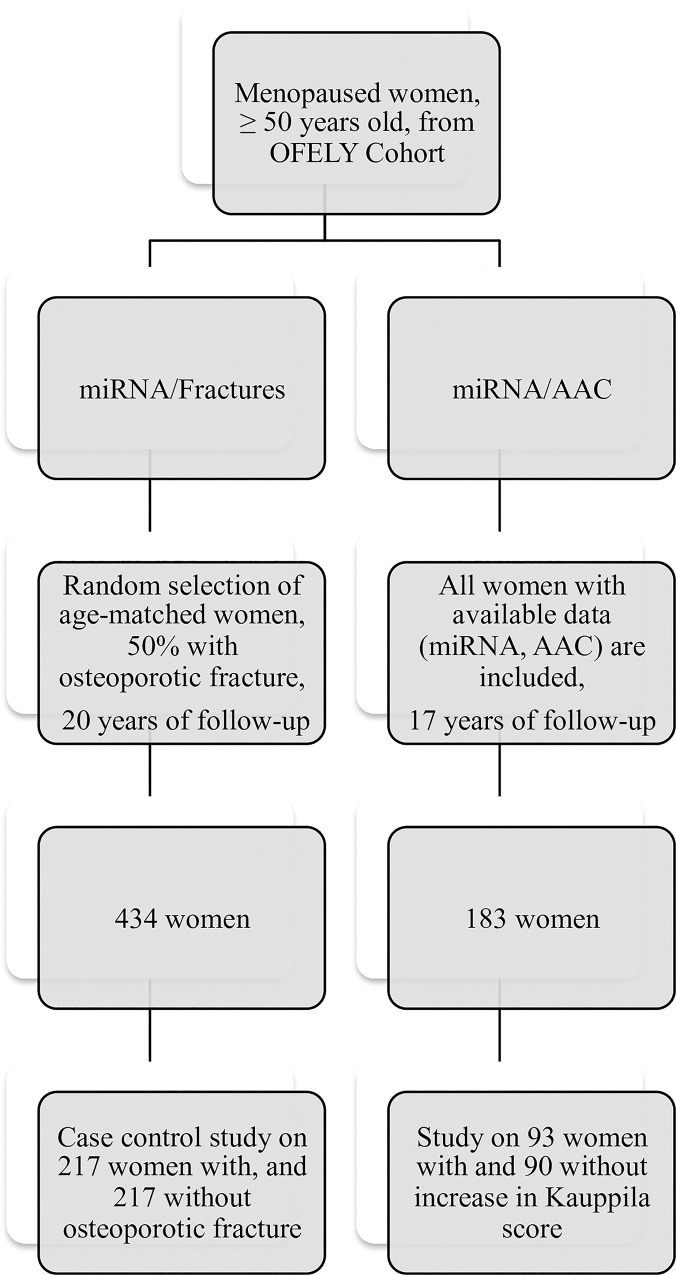
Study design.

We conducted two studies in women from the OFELY cohort (Fig 1). The OFELY cohort (Os des FEmmes de LYon) is an ongoing prospective study of the determinants of bone loss [28]. It included 31–89 year-old women, recruited between February 1992 and December 1993, and randomly selected from the affiliates of a French health insurance company (Mutuelle Générale de l’Education Nationale) with an annual follow-up. Available blood samples at inclusion, radiographic scores of AAC at inclusion and at year 17 and the knowledge of incident fractures in the course of 20 years were selection criteria of these post-menopausal women.

To study the association between miRNA expression and incident osteoporotic fracture, we conducted a case control analysis with 217 women with incident fragility fracture and 217 age-matched women without incident fragility fracture over 20 years. To study the link between miRNA expression and the progression of AAC, we conducted an analysis among the 183 women with available data of AAC over 17 years. Written informed consent was obtained from each participant and the study was approved by the local ethics committee (Comité de Protection des Personnes Sud-Est II).

### Clinical parameters

Women completed a written health questionnaire at baseline, including medical history, tobacco use, medication use, fall(s) in the course of the past 12 months, and occurrence of radiologically confirmed low-trauma fractures [29]. Height and weight were measured and body mass index (BMI) was calculated (kg/m2).

Moreover, bone mineral density (BMD) was measured by DXA with a QDR 2000 device (Hologic, Waltham, MA, USA) at the lumbar spine and the total hip at baseline. The T-score was calculated from the HOLOGIC France references values.

### Blood sample collection and biochemical measurements

Blood samples were collected between 8:00 and 9:30 a.m. after an overnight fast. Serums were aliquoted in 1mL tubes and then stored frozen at –80°C until assayed. The macroscopical analysis at time of blood sampling assessed for serum quality with none of the serum affected by haemolysis. Only two serums had fibrin at time of extraction. At inclusion time, a number of parameters were measured in morning fasting blood samples including, Osteocalcin (ng/ml), N-terminal propeptide of type I collagen (PINP) (ng/ml), Bone alkaline phosphatase (BAP) (ng/ml), C-telopeptide of type I collagen (sCTX-I) (ng/ml), Calcium (mg/l), Phosphate (mg/l), Creatinine (mg/l), globular filtration rate (GFR MDRD) (ml/min/1.73m^2^), 25-hydroxycholecalciferol (25-OH Vitamin D) (ng/ml) and parathyroid hormone (PTH) (pg/ml). The bone turnover markers (Osteocalcin (ng/ml), PINP (ng/ml) and sCTX-I (ng/ml)) were measured in serum by automated tests (Elecsys N-MID osteocalcin, Elecsys PINP and Elecsys b-Crosslaps, respectively; Roche Diagnosis, Meylan, France). BAP (ng/ml) was measured by Elisa (Metra BAP EIA kit, Quidel Corporation, USA). Creatinine was measured, and GFR MDRD was calculated using a modified kinetic method and the Chronic Kidney Disease Epidemiology Collaboration, respectively [30]. 25-OH Vitamin D (ng/ml) was assessed by radioimmunoassay (DiaSorin, Stillwater, USA) after acetonitrile extraction. PTH (pg/ml) was measured using a human specific two-site immunochemiluminescence assay (ELECSYS, Roche, Indianapolis, IN, USA). Calcium (mg/l) and Phosphate (mg/l) were measured by colorimetric assay.

### Fracture evaluation

Incident non-vertebral and clinical vertebral fragility fractures, all confirmed by radiographs or surgical report were reported at every annual follow-up. For women who did not come to the Clinic for their annual evaluation, a letter was sent annually to identify the occurrence of any fracture. Only low trauma fractures (i.e., those occurring as a result of falls from standing height or less) were taken into account in this analysis, and we excluded fractures of fingers, toes, skull, and face. Vertebral fractures were also assessed in women aged 50 years and over at the inclusion in the study confirmed on lateral X-ray films of thoracic and lumbar spine at baseline and every 4 years, and after 19 years on DXA (Hologic Discovery, HOLOGIC Inc; Bedford, MA) using Vertebral Fracture Assessment software (VFA). They were identified with the semiquantitative method of Genant et al [31] by a trained physician (ESR).

### Assessment of abdominal aortic calcification

AAC were measured on lateral spine radiographs using the Kauppila score. The Kauppila score is a 24-point semi quantitative score, used to quantify the presence and severity of AAC. Calcifications are scored (0 to 3 points) on the 8 segments of the aortic abdominal wall according to their length, and these scores are summed to obtain a 24-point score [32].

The score was evaluated at inclusion and after 17 years of follow-up by a single reader (MEP). Reproducibility was assessed using 30 radiographs and calculated using the intraclass correlation coefficient (ICC). The intra-reader reproducibility was excellent (ICC = 0.968, IC 95% [0.935–0.984]). The inter-reader agreement was assessed, with a reader (PS) trained to AAC for Kauppila scoring. It was also excellent (for the 1^st^ scores ICC = 0.967, 95%CI: 0.933–0.984 and for the 2^nd^ scores ICC = 0.953, 95%CI 0.906–0.977).

A methodology of missing Kauppila score data was established. As AAC do not decalcify, unavailable data were replaced by “0” if the Kauppila score at subsequent follow-up was 0. Data were considered unavailable if the radiographs were missing or low quality. Radiographs were considered of good quality if the X-ray projection was the one expected (lateral lumbar spine radiograph), if the lower endplate of T12 and the superior endplate of S1 were viewable, and if the distance in front of the anterior vertebral wall was sufficient. In the absence of AAC, this distance was considered sufficient if the measure was at least of 2.5 cm from the middle of the vertebral body, for an anteroposterior length of the vertebral body of 4 cm. In the presence of AAC, radiographs were considered of low quality if the anterior vascular wall was not visible in its entirety from L1 to L5.

An increase of >1 point in Kauppila score (Δ Kauppila >1) was retained to define AAC aggravation over 17 years.

### Quantification of circulating miRNAs

#### Preselection of miRNAs

To identify candidate miRNAs linked with increased risk of AAC aggravation and incident osteoporotic fracture, a first miRNA selection was done by computational prediction of their target genes using TargetScan and from experimentally validated target genes using MiRWalk database. From this, three miRNAs (miRs-26a-5p, 34a-5p, and 223-5p) were selected based on their known regulatory functions in vascular calcification and bone metabolism and on previously reported experimental animal research and clinical trials (Table 1).

**Table 1:** Potential activity of candidate microRNAs in bone-related disease and aortic calcification.

MiRs	Target gene* (nucleotide site in 3’UTR)	Regulatory function	*In vitro* and animal study	Clinical relevance	Ref
miR-26a-5p	-*CTGF* (38–44 ; 7 merA1) -*BMP/SMAD1* (46–53 ; 8mer) (103–109 ; 7merA1)	↓ in vascular diseases angiogenesis, myocardial infarct size and improve heart function ↓ osteoclastic differentiation	↓ in rat cardiac hypertrophy ↓ vascular smooth muscle cell calcification ↓ calcification related-genes (BMP2, SMAD1,2, ALP)	↓ in aortic valve calcification and stenosis	[33–37]
miR-34a-5p	-*PPP1R10* (822–828 ; 7–8 mer) -*TGIF2* (90–97 ; 8 mer) -*JAG1* (1302–1308 ; 7–8 mer)	↑Age-induced cardiac cell death ↓Osteoclastogenesis ↓osteoblast differentiation	↑ cardiac dysfunction after myocardial infarction ↓ bone resorption, bone mass ↓ *in vivo* bone formation of human stromal stem cells.	Antagomir therapeutic improves aging cardiac dysfunctions and stimulates osteoblast differentiation Attenuates osteoporosis ↓ Therapeutic strategy for enhancing bone formation	[38–41]
miR-223	-*SCARB1* (575–582 ; 8 mer) *-FCGR1A* (141–147 ; 7–8 mer) -*NFIA* (811–818 ; 8 mer)	↓ HDL-cholesterol uptake, biosynthesis, cholesterol efflux ↓proliferation and ↑ apoptosis in vascular wall ↑ Inorganic Phosphate-induced- osteoclastogenesis and osteoclast differentiation	↑HDL-cholesterol, hepatic and total plasma cholesterol in KD mice ↑ in serum and atherosclerotic vascular walls ↓atherosclerotic lesions in mice ↓levels in serum, blood cells of chimeric-KD-mice Modulate the balance osteoblast-like/ osteoclast-like	Biomarker for altered cholesterol homeostasis ↑levels in serum from Kawasaki disease ↓ in serum from patients with chronic kidney disease Treatment to reverse vascular calcification without altering bone structure in CKD-MBD Provide new therapeutic target for atherogenesis	[42–47]

*: The target genes are indicated with the nucleotide position of the seed region in the 3’-UTR (according to Target Scan). ↑ ↓ are for up- and -down regulation respectively.

#### MiRNA quantification

Total RNA was extracted from 200μl serum with the miRCURY Biofluids extraction kit of (Exiqon) according to manufacturer recommendations. Samples were thawed on ice and centrifuged at 3,000g for 5 minutes. A lysis buffer solution containing 1μl of glycogen as RNA carrier and a synthetic spike-in control RNA (cel-miR-39-3p) as internal control was added to the serum. After cellular lysis and protein precipitation, supernatant was placed on a silica micro-column and treated with rDNAse. RNA was eluted with 40 μl RNAse/DNAse-free water (Invitrogen) and stored at -80°C.

MiRNAs were quantified by TaqMan Advanced miRNA technology (Applied Biosystems, ThermoFisher Scientific). The first analytical step consists of the universal reverse transcription (RT) to prepare cDNA from 2μl of total RNA using a TaqMan Advanced miRNA cDNA synthesis kit. Briefly, the kit uses 3' poly-A tailing and 5' ligation of an adaptor sequence for each end extension of the mature miRNAs present in the sample, prior to RT. Universal RT primers anneal to the universal sequences present on both the 5' and 3' extended ends of the mature miRNAs. Quantification of miRNAs expressed at low copy number was improved by 14 cycles of cDNA amplification in a 2X TaqMan PreAmp master mix containing Megaplex PreAmp primers.

The qPCR amplification was performed on 1:10 dilution of cDNA obtained by miR-Amp reaction on 5 μl of the RT reaction, using the 2X Fast Advanced Master Mix and the 20X TaqMan Advanced miRNA Assays. These TaqMan Advanced miRNA assays (Table 2) contain pre-formulated primers and TaqMan MGB (minor groove binder) probes that allow the recording of fluorescence signal in the PCR reaction. The MicroAmp Fast Optical 96-Well Reaction Plates (ThermoFisher) were designed for the quantification in duplicate of seven miRNAs on the QuantStudio 7 flex (Applied Biosystems) according to the manufacturer’s protocol. The C_T_ (threshold cycle value) was recorded as the cycle number at which the fluorescence generated within a reaction crosses the fluorescence threshold, a fluorescent signal significantly above the ROX fluorescence background recorded in each sample [48].

**Table 2:** Identification of the candidate microRNAs, of the three endogenous normalizer microRNA used and of the spike quality control of the analysis.

miR Base ID	NCBI Accession Number	TaqMan Advanced miRNA Assay (ID)	Sequence of the mature miRNA 5’————————— 3’
hsa-miR-26a-5p	MIMAT0000082	**477995_mir**	UUCAAGUAAUCCAGGAUAGGCU
hsa-miR-223-5p	MIMAT0004570	**477984_mir**	CGUGUAUUUGACAAGCUGAGUU
hsa-miR-34a-5p	MIMAT0000255	**rno481304_mir**	UGGCAGUGUCUUAGCUGGUUGU
hsa-miR-191-5p	MIMAT0000440	**477952_mir**	CAACGGAAUCCCAAAAGCAGCUG
hsa-miR-222-3p	MIMAT0000279	**477982_mir**	AGCUACAUCUGGCUACUGGGU
hsa-miR-361-5p	MI0000760	**481127_mir**	UUAUCAGAAUCUCCAGGGGUAC
cel-miR-39-3p	MI0000010	**478293_mir**	UCACCGGGUGUAAAUCAGCUUG

All the nomenclature is according to miRBase V21 and the TaqMan Advanced miRNA Assays are from Applied Biosystems.

The miRNA level was expressed by relative quantification according the following formula: Relative quantification = 2^–ΔΔCT^, with ΔC_T_ = (C_T_ miRNA–C_T_ mean of the 3 endogenous controls) and ΔΔC_T_ = (ΔC_T_ of the miRNA –ΔC_T_ mean of the miRNA through all samples). Data were normalized with the mean of expression level of three endogenous miRNAs: hsa-miR-191-5p, hsa-miR-222-3p and hsa-miR-361-5p, that are known to be ubiquitously expressed and without reported impact on cardiovascular diseases or bone. The exogenous spike cel-miR-39-3p was used as a qPCR quality control (Table 2).

#### Statistics

The baseline characteristics of the women are presented as mean ± SD for continuous variables and proportion of patients (%) for categorical variables. Chi-squared tests and Wilcoxon tests were used to compare women with and without incident fracture. In order to identify the association between miRNAs and the risk of incident fracture or the aggravation in Kauppila score, we performed a logistic regression using quartiles of relative quantification for each miRNA.

All results are expressed in median [interval interquartile] or mean +/- SD. All statistical analyses were performed using Stata 12 (StataCorp LP, College Station, Texas, USA).

## Results

### Clinical characteristics

In the first study, we have selected the 217 women who had incident fragility fracture over 20 years of follow-up and age-matched them with a random sample of non fractured women (63 [57–72] years old). Fractured women were significantly thinner than controls (body weight = 59.2 [52.8–64.4] vs 60.6 [54.6–67.6] kg, p = 0.04; and BMI = 23.2 [21.3–25.5] vs 24.2 [22–27.2] kg/cm^2^, p = 0.006) (Table 3).

**Table 3:** Baseline demographic and biological characteristics of cases (with incident fracture) versus controls (without incident fracture), and miRNA quantification (median [interval interquartile]).

	Cases (n = 217)	Controls (n = 217)	P values
Age (years)	63 [57–72]	63 [57–72]	0.93
Weight (kg)	59.2[52.8–64.4]	60.6 [54.6–67.6]	**0.04**
Height (cm)	158 [154–162]	158 [154–162]	0.50
BMI (kg/m2)	23.2 [21.3–25.5]	24.2 [22–27.2]	**0.006**
Total Hip T-score	-1.7 [-2.3- -1.0]	-1.16 [-2.0- -0.3]	**<0.0001**
Lumbar Spine T-score	-1.9 [-2.6- -1.1]	-1.0 [-1.8- -0.3]	**<0.0001**
Smokers, n(%)	17 (8)	14 (6)	0.58
Fallers in the past year, n(%)	75 (35)	88(41)	0.20
Prior Fx, n(%)	32(15)	44(20)	0.13
Osteoporosis treatments, n(%)^a^	36(17)	39(18)	0.70
Comorbidity, n(%)^b^	50(23)	42(19)	0.35
Cardiovascular diseases, n(%)	45(21)	39(18)	0.47
Osteocalcin (ng/ml)	12.7 [10.2–16.2]	12.1 [10.0–15.6]	0.29
PINP (ng/ml)	45.7 [34.6–60.5]	45.1[33.0–64.1]	0.88
Bone BAP (ng/ml)	11.8 [9.4–14.7]	12.2 [9.6–15.0]	0.33
sCTX-I (ng/ml)	0.51 [0.3–0.6]	0.46 [0.3–0.7]	0.27
Calcium (mg/l)	93 [91–95]	93 [91–95]	0.20
Phosphate (mg/l)	34 [32–37]	34 [32–37]	0.28
Creatinine (mg/l)	9.4 [8.5–10.2]	9.3 [8.7–10.3]	0.78
GFR (MDRD) (ml/min/1.73m^2^)	63.9 [58.2–71.9]	64.2 [58.0–71.1]	0.82
25-OH Vitamin D (ng/ml)	32 [23–41]	34.5 [23.5–45.5]	0.24
PTH (pg/ml)	31.6 [23.1–39.3]	29.8 [24.2–39.8]	0.84
miRNA (Relative quantification)			
miR-26a-5p	1.06 [0.85–1.27]	0.99 [0.85–1.17]	0.07
miR-34a-5p	1.15 [0.53–1.87]	1.26 [0.60–2.07]	0.35
miR-223-5p	1.01 [0.68–1.43]	1 .05 [0.72–1.56]	0.32

^a^ hormonal replacement therapy

^b^ Diabetes mellitus, thyroid or parathyroid disorders, renal insufficiency, rheumatoid arthritis, gastric or intestinal surgery, malignancy and stroke

In the second study, 183 women were included to examine the link between the aggravation in AAC and the miRNA expression, 93 with and 90 without an aggravation in Kauppila score during the 17 years of follow-up (respectively 58 [55–61] and 55 [53–58] years old, respectively). Weight and BMI were not significantly different between both groups (Table 4).

**Table 4:** Baseline demographic and biological characteristics of 183 women with versus without an aggravation in Kauppila score during 17 years of follow-up (median [interval interquartile]) and miRNA quantification (median [interval interquartile]).

	Aggravation in Kauppila score (n = 93)	No aggravation in Kauppila score (n = 90)	P value
Age (years)	58 [55–61]	55 [53–58]	**<0.0001**
Weight (kg)	56.8 [51.4–63.6]	56.6 [52.2–60.6]	0.79
Height (cm)	158 [156–162]	160 [156–163]	0.23
BMI (kg/m2)	22.5 [20.9–24.9]	22.3 [20.9–23.7]	0.36
Total Hip Tscore	-1.2 [-1.9- -0.5]	-1.1 [-1.6- -0.4]	0.47
Lumbar Spine Tscore	-1.5 [-2.4- -1.1]	-1.5 [-2.1- -0.2]	0.27
Smokers, n(%)	8(9)	3(3)	0.13
Fallers in the past year, n(%)	25(27)	21 (23)	0.58
Prior Fracture, n(%)	2(2)	1(1)	0.58
Osteoporosis treatments, n(%)^a^	26(28)	33(37)	0.21
Comorbidity^b^, n(%)	20(19)	22(23)	0.77
Cardiovascular diseases, n(%)	9(10)	5(6)	0.29
Initial Kauppila score >0, n(%)	36 (39)	6(7)	**<0.0001**
Osteocalcin (ng/ml)	11.8 [10.1–14.8]	12.6 [9.5–15.8]	0.41
PINP (ng/ml)	46.4 [35.9–57.3]	46.7[35.6–62.4]	0.49
Bone AP (ng/ml)	11.8 [8.7–13.9]	11.7 [10.0–14.4]	0.53
sCTX-I (ng/ml)	0.51 [0.3–0.7]	0.52 [0.3–0.6]	0.78
Calcium (mg/l)	93 [91–95]	93 [91–95]	0.28
Phosphate (mg/l)	35 [32–37]	33 [31–37]	0.25
Creatinine (mg/l)	9.2 [8.5–10.1]	9.3 [8.6–9.8]	0.57
GFR (MDRD) (ml/min/1.73m^2^)	66.7 [59.5–72.5]	66.8 [61.3–72.6]	0.36
25-OH Vitamin D (ng/ml)	38 [28–47]	38 [29–45]	0.81
PTH (pg/ml)	28.1 [23.1–37.8]	28.6 [22.7–35.4]	0.65
miRNA (Relative quantification)			
miR-26a-5p	1.09 [0.94–1.28]	1.10 [0.89–1.30]	0.95
miR-34a-5p	0.78 [0,46–1.21]	0.73 [0.38–1.50]	0.90
miR-223-5p	0.97 [0.69–1.22]	0.78 [0.56–1.22]	0.11

^a^ hormonal replacement therapy

^b^Diabetes mellitus, thyroid or parathyroid disorders, renal insufficiency, rheumatoid arthritis, gastric or intestinal surgery, malignancy and stroke

### Associations between miRNA expression and incident fracture

Levels of miR-26a-5p, miR-34a-5p and miR-223-5p in patient serum were not significantly different between women with or without incident fracture (Table 3).

The increase in one quartile of miRNA quantification was not associated with the risk of incident fracture, with or without adjustment for BMI and Total Hip BMD (adjusted OR (95%CI) for miR-26a-5p: 1.18 (0.99–1.41); miR-34a-5p: 0.88 (0.73–1.05); miR-223-5p: 0.98 (0.82–1.17)).

### Associations between miRNA expression and aggravation of AAC

The Kauppila score increased from 0 [0–0] to 2 [0–5] over17 years.

Women with AAC aggravation were significantly older, by about 3 years (p<0.0001). MiR-26a-5p, miR-34a-5p and miR-223-3p were not significantly linked to the aggravation in AAC (Table 4). The increase in one quartile of miRNA quantification was not associated with AAC aggravation, with or without adjustment for age and Kauppila score at baseline (adjusted OR (95%CI) for miR-26a-5p: 0.93(0.69–1.27); miR-34a-5p: 0.97 (0.71–1.34); miR-223-5p: 1.13(0.81–1.57)).

## Discussion

This analysis from the OFELY cohort showed that the serum concentrations of three microRNAs involved both in the regulation of bone turnover and vascular calcification were not significantly associated with incident fractures and progression of AAC.

Studies from our group [3,15,49] and others [11–14,50] have previously demonstrated the association of AAC with bone fragility, two processes that share common pathogenetic mechanisms in men and women from various populations, especially in the elderly [51,52]. More specifically, in postmenopausal women, the aggravation in AAC score is linked to the increase in bone resorption in particular when BMD is inversely linked to AAC score [5,12,13]. The connection of AAC with poor bone status, higher risk of fracture and bone loss has been consistently reported in cohorts after adjustment for numerous shared risk factors, mainly age, lifestyle, co-morbidity, hormones, vitamin D status and medications that influence both pathologies [53]. For example, severe AACs are linked to a greater prevalence of vertebral fracture after adjustment for age and BMI, and a greater number of vertebral fractures has been shown in older men, after adjustment for BMD and other variables [15].

Epigenetic factors interfere with the “calcic paradox”, a common determinant of AAC and bone fragility where cardiac valves calcify and bone decalcifies. At the transcriptomic level, the calcification of the aortic media is regulated by calcium deposal, inorganic phosphate and bone-related factors. In this study we looked for epigenetic factor as a single assessment of AAC and fracture risk that could identify patients for prevention and treatment of cardiovascular and bone disorders. Therefore, we have designed the study to assess the risk factors that are shared by both conditions. Two groups of patients have been selected in the well-characterized OFELY cohort: a sizeable control sample of women differing significantly only for incident fracture and a second group of women with available data to explore AAC over 17 years of follow-up. Secondly, we used miRNA level in serum as an easily accessible epigenetic marker to assess the dysregulation of gene expression in both diseases.

Aberrant miRNA signaling alters bone homeostasis and contributes to the progression of bone disorders. Among these, miR-26a-5p, 34a-5p, 125b-5p, 145-5p, 146a-5p, 221-5p and 223-5p regulate the transformation of smooth muscle to vascular cells and participate to calcification by interacting with osteoblasts and osteoclasts [22,23,33,34]. Therefore, based on their mRNA targets, known regulatory function and clinical relevance, we have selected miR-34a-5p, miR-26a-5p and miR-223-5p as common potential biomarkers of these diseases.

MiR-26a-5p is known to alter pathological angiogenesis by targeting BMP/SMAD 1 signaling (bone morphogenic protein/SMAD1) [35] and impaired angiogenesis in diabetic dermal wound healing [36]. Administration of miR-26a-5p inhibitor to mice increases SMAD1 expression, induces angiogenesis, reduces myocardial infarct size, and improves heart function [37]. Aortic stenosis decreased miR-26a-5p expression in patients undergoing aortic valve replacement compared to those with aortic insufficiency, suggesting a decrease in valvular aortic calcification. Although the impact of miR-26a-5p on bone itself is poorly reported, CTGF expression and BMP/SMAD1 pathway are involved in osteoclastic differentiation and these pathways also impact on osteoblastogenesis [19,35]. *In vitro*, miR-26a-5p inhibited vascular smooth muscle cell calcification by regulating the expression of CTGF, OPG, RANKL and ALP [34]. MiR-34 family has been consistently reported to interfere with cellular regulation of bone homeostasis. Mir-34a decreased osteoclastic differentiation by targeting several pathways including NFATc1 or Tgif2 [38]. Transgenic mice overexpressing miR-34a had a lower bone resorption and higher bone mass while miR-34a knock out in mice showed higher bone resorption and reduced bone mass. Other studies, in triple knock-out mice for miR-34a, b and c confirmed that the loss of function of miR-34 induces higher bone resorption [39]. The overexpression of miR-34a inhibited osteoblastic differentiation of human stromal stem cells, and conversely, *in vivo* bone formation was increased in case of miR-34a deficient human stromal stem cells [40], with JAG-1, a ligand for Notch 1 as a potential target of miR-34. By inhibiting osteoblast differentiation and *in vivo* bone formation, miR-34a is a major actor of the calcic paradox. However, on the cardiac site, age is a crucial determinant of miR-34a activity since cardiac expression of miR-34a increases with age in mice. MiR-34a deficient mice had lower age-induced apoptosis of cardiomyocytes and a better cardiac function [41] and miR-34a inhibition was linked to lower cell death and fibrosis after acute myocardial infarction, with improvement of myocardial function recovery. One of the pathways suggested is PNUTS (protein phosphatase 1 (PP1) nuclear targeting subunit) also known as PPP1R10, with a reduction of telomere shortening and age-associated DNA damage responses [41]. In our study we found abundant levels of the miR-223 mature forms in serum, of the initially described miR-223* (-3p) and of its hairpin counterpart, miR-223-5p. This high miRNA expression would improve the specificity and reproducibility of their measurement in serum. This increased the potential of miR-223, that has been associated to inflammation, atherogenesis, vascular calcification, cardiovascular and bone disease, to be a biomarker in these pathologies [33,42,43]. The genetic ablation of miR-223 in mice increased HDL-cholesterol and plasma total cholesterol by coordinating transcriptional control of several genes in lipoprotein and cholesterol metabolism [44]. MiR-223 expressed in bone marrow-derived blood cells is secreted in serum and enters the endothelial and vascular smooth muscle cells to act as an endocrine genetic signal in vascular injury. It may provide a novel mechanism for vascular complication in Kawasaki disease [45]. Although the role of miR-223 in osteoclastogenesis is controversial, its expression is affected during osteoclastogenesis [46]. Inorganic phosphate decreased the miR-223 level and osteoclastogenesis by affecting the expression of NFIA and RhoB in human osteoclasts and calcifying vascular smooth muscle cells. In chronic kidney disease, miR-223 increased in serum patients, reflecting changes of bone and calcification pathways [47].

Circulating mir-26a-5p, miR-34a-5p and miR-223-5p were not significantly different between women with or without incident fracture. A difference in the clinical characteristics of our cohort might explain the discrepancy between these results and previous studies [3,25,54,55]. Here, the study was designed to include 434 age-matched women with and without fractures during the 20 years of follow-up and to eliminate the confounding factors that may not be related to the miRNA influence in the two pathologies. The level of serum miRNA is associated with aging in a wide range of physiological processes and diseases including sarcopenia [55]. In our first study, cases and controls do not differ by parameters such as bone markers, calcium, phosphate, PTH and 25-OH vitamin D. The two groups differed significantly in BMI and weight but the associations were the same after BMI-adjustment. We hypothesize that biomarker studies associating circulating miRNA levels to bone fragility and fractures that are reported in smaller sample sizes of patients did not exclude some of these confounding factors.

Circulating miR-26a-5p, miR-34a-5p and miR-223-5p were not significantly linked to AAC aggravation over 17 years and age-adjusted OR was not significant either. None of these miRNAs was associated to the aggravation of Kauppila score in comparable size samples of women that experienced similar comorbidity, cardiovascular disease and prior fractures. The weakness of our study may rely on the evaluation of AAC with standard radiographs, as radiographs are less sensitive than other methods like computed tomography (CT) that may detect smaller calcifications. Using quantitative CT, AAC may be evaluated with the Agatston score that relies on the calcic density of the vascular wall, giving a more precise evaluation than standard radiographs. This method, however, delivers more radiation, is more expensive and more difficult to use in daily practice. We have also reported that the severity of AAC is positively associated with vertebral fracture in older men [15]. Prospective studies confirm the association between baseline AAC severity and prospectively assessed fracture risk in both sexes [3]. In the present study, women presented low Kauppila scores, and a mild aggravation on 17 years of follow-up. Women included here might not be a representative sample to study the association of cardiovascular events with fracture. We have also to consider that the serum concentration of these three miRNAs might not adequately reflect miRNA-induced epigenetic regulation taking place at cellular level in bone and vascular wall [55]. Although serum utilization is suitable for clinical research, miRNA secreted by cells and tissue microenvironment induce changes in miRNA profiling that are confined to extracellular vesicles such as exosomes and lipoproteins, when released into the blood stream [55,56]. Subtle modification within the bulk of miRNAs might be difficult to assess from an analytical point of view, limiting use in clinical settings.

In conclusion, serum concentrations of three selected miRNAs, miR-26a-5p, -34-5p and -223-5p were not associated with incident fractures nor with AAC aggravation in a large sample of women from the OFELY cohort. These miRNAs are unlikely to become biomarkers of clinical use.

## Abbreviations

AAC: Abdominal Aortic Calcification
AP: Alkaline Phosphatase
BMD: Bone Mineral Density
BMP/SMAD1: Bone Morphogenetic Protein/ SMAD1
BMI: Body Mass Index
cDNA: Complementary Deoxyribonucleic Acid
C_T_: Cycle Threshold
CTGF: Connective Tissue Growth Factor
CTX-I: Carboxy-terminal Cross-linking Telopeptide of Type I Collagen
DXA: Dual Energy X-ray Absorptiometry
GFR (MDRD): Glomerular Filtration Rate (Modification of Diet in Renal Disease)
ICC: Intraclass Correlation Coefficient
JAG-1: Jagged 1
miRs: miRNAs, microRNAs
NFATc 1: Nuclear Factor of Activated T-cells, cytoplasmic 1
NFIA: Nuclear Factor I A
OFELY: Os des FEmmes de Lyon
OPG: Osteoprotegerin
OR: Odds Ratio
PCR: Polymerase Chain Reaction
PINP: Procollagen Type I amino-terminal Propeptide
PNUTS: PP1 Nuclear Targeting Subunit
PTH: Parathormone
RANKL: Receptor Activator of Nuclear Factor-kB
RNAs: Ribonucleic Acid
RT: Reverse Transcription
SD: Standard Deviation
Tgif2: Thymine Guanine-Interacting Factor 2
VFA: Vertebral Fracture Assessment
